# Sustainable stability-indicating spectra manipulations for the concurrent quantification of gliclazide and its impurity A as the possible alkaline degradation product: Multi-Color Analytical Profile Assessment

**DOI:** 10.1038/s41598-026-67045-4

**Published:** 2026-08-22

**Authors:** Ebraam B. Kamel, Ahmed Elsayed Abdelmegeed, Hanaa Saleh, Magda Elhenawee, Eman A. Bahgat

**Affiliations:** 1 Pharmaceutical Chemistry Department, Faculty of Pharmacy, Egyptian Russian University, Badr City, Cairo Egypt; 2 Pharmaceutical Analytical Chemistry Department, Faculty of Pharmacy, Zagazig University, Zagazig, Egypt

**Keywords:** Gliclazide, Stability-indicating method, Green analytical chemistry, First derivative spectrophotometry, Fourier self-deconvolution, Greenness assessment, Chemistry, Environmental sciences

## Abstract

A green, stability-indicating UV-spectrophotometric methods were designed and optimized for the quantitative determination of gliclazide (GLI), a second-generation sulfonylurea widely used in the management of type 2 diabetes mellitus (T2DM). The methods integrate first-derivative spectrophotometry (D^1^) and Fourier Self-Deconvolution (FSD) to resolve the extensive spectral overlap between GLI and its alkaline degradation product (Gliclazide Impurity A) without prior separation. The assay demonstrated excellent linearity within 2.5–25 µg/mL for GLI and 2.5–15 µg/mL for its degradation product. LOD ranged from 0.31 to 0.43 µg/mL and LOQ from 0.94 to 1.31 µg/mL for both analytes. Using ethanol as a green, non-toxic solvent further enhanced the method’s environmental sustainability. Method validation, performed according to ICH Q2(R1) guidelines, confirmed its selectivity, reproducibility, robustness, and accuracy with mean recoveries of 99.48 ± 0.83 and 100.87 ± 1.52 for GLI in the D^1^ and FSD methods, respectively. Comprehensive forced degradation studies under alkaline stress verified the procedure’s stability-indicating capability, enabling reliable quantification of GLI in the presence of its degradation products. The greenness evaluation using Analytical Eco-Scale, GAPI, AGREE, NEMI tools, EPPI tool, AGSA tool, and MOGAPI method collectively affirmed the method’s eco-friendly and sustainable nature. Furthermore, the analytical strategy was comprehensively assessed using multi-dimensional tools, including RAPI and AMRI for analytical performance and reliability, CaFRI for carbon-footprint reduction, CACI for analytical practicality, and the RGB 12 model for an integrated multi-colored analytical profile. In summary, the developed methods provide a highly efficient, environmentally friendly, and analytical alternative to conventional chromatographic techniques for the routine quality control and green pharmaceutical analysis of gliclazide in bulk and dosage forms.

**Supplementary Information:**

The online version contains supplementary material available at 10.1038/s41598-026-67045-4.

## Introduction

Gliclazide is a second-generation sulfonylurea oral hypoglycemic agent, first introduced in the 1970 s for the treatment of type 2 diabetes mellitus (T2DM)^1^. It exerts its antidiabetic effect by stimulating insulin secretion from pancreatic β-cells through closure of ATP-sensitive potassium channels. It also exhibits additional vasculoprotective and antioxidant properties^2^. These pleiotropic effects make gliclazide particularly suitable for patients with cardiovascular risk factors.

**Fig. 1:**
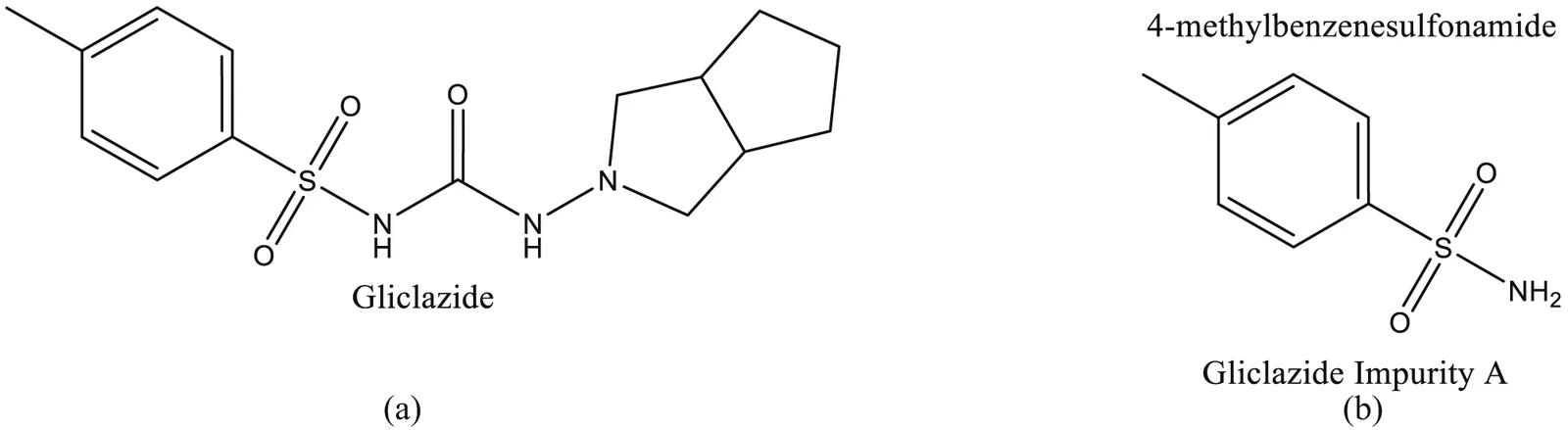
Chemical structure of (**a**) gliclazide and its (**b**) official impurity (Impurity A). The chemical structures were drawn using ChemDraw Professional, version [16.0] (PerkinElmer Informatics, USA). Available at: https://revvitysignals.com/products/research/chemdraw.

From a physicochemical standpoint, gliclazide (C₁₅H₂₁N₃O₃S), Fig. 1(a), is a white to slightly off-white crystalline compound exhibiting poor aqueous solubility yet demonstrating appreciable solubility in ethanol and dimethylformamide. It has a molecular weight of 323.41 g/mol and contains a characteristic sulfonylurea moiety, which renders the molecule chemically labile under alkaline conditions due to its susceptibility to hydrolytic degradation^3^. Herein, the alkaline stress conditions yielded gliclazide impurity A, Fig. 1(b).

The stability of gliclazide represents a critical factor in pharmaceutical formulation, as sulfonylurea derivatives are particularly susceptible to alkaline hydrolysis degradation. Such degradation pathways can compromise the drug’s potency, efficacy, and safety, underscoring the importance of developing stability-indicating analytical methods for its reliable assessment and quality control^4^. Therefore, stability-indicating analytical methods (SIAMs) are essential to distinguish the intact drug from its degradation products, ensuring the quality and shelf life of pharmaceutical preparations^5–8^.

Preliminary stress testing under acidic, thermal, and photolytic conditions revealed no significant or reproducible degradation behavior compared to alkaline hydrolysis. Therefore, alkaline conditions were selected as the primary degradation pathway for this study^9,10^.

In recent years, Green Analytical Chemistry (GAC) has attracted significant attention, driven by the global emphasis on environmental sustainability and resource conservation. Although traditional chromatographic techniques are highly effective and widely adopted, they typically require large quantities of toxic organic solvents, consume significant energy, and generate substantial chemical waste. In contrast, green spectrophotometric approaches, particularly those employing eco-friendly solvents such as ethanol, present sustainable, cost-effective, and safer alternatives that maintain analytical accuracy and sensitivity while reducing environmental impact^11^.

Several analytical methods have been reported for the determination of gliclazide in bulk and pharmaceutical dosage forms, including spectrophotometric^12–14^, HPLC^15–18^, LC-MS/MS^19^, capillary electrophoresis^20^, and electrochemical methods^21,22^. Although sensitive, these methods are often costly, time-consuming, and resource-intensive. In contrast, UV-spectrophotometry remains a simple, cost-effective, and widely accessible technique. However, limited studies have explored the use of first derivative (D¹) and Fourier Self-Deconvolution (FSD) approaches in a stability-indicating context for gliclazide. The current work overcomes these drawbacks by introducing simple, eco-friendly spectral manipulation techniques that efficiently resolve GLI and Impurity A without any separation step, while employing inexpensive green solvents, minimal energy consumption, and straightforward instrumentation. This positions the proposed methods as practical alternatives for routine quality-control applications.

Furthermore, the evaluation of greenness in analytical procedures through modern assessment tools such as the Green Analytical Procedure Index (GAPI), Analytical Eco-Scale, National Environmental Methods Index (NEMI), and Analytical GREEnness (AGREE) metric remains insufficiently incorporated into many existing gliclazide-related studies. This deficiency highlights the urgent need for analytical methodologies that integrate both scientific rigor and environmental responsibility. Accordingly, the present work aims to develop and validate a simple, eco-conscious, and stability-indicating UV-spectrophotometric method for the quantitative determination of gliclazide in the presence of its alkaline degradation products. The proposed approach utilizes first-derivative spectrophotometry (D¹) and Fourier Self-Deconvolution (FSD) techniques to achieve spectral resolution without prior separation. Ethanol, a green and low-toxicity solvent, serves as the analytical medium, while the method’s environmental sustainability is systematically evaluated using four complementary greenness assessment tools, GAPI, NEMI, Eco-Scale, and AGREE. The novelty of this work lies in the integration of FSD with D¹ spectroscopy for gliclazide stability analysis, the use of ethanol as a sole solvent in a fully green spectrophotometric procedure and comprehensive greenness assessment using four validated tools, filling a gap in current literature.

The novelty of the present work does not reside in the individual analytical tools themselves, but rather in their synergistic integration to address a specific unresolved analytical challenge, namely the selective determination of gliclazide in the presence of its official alkaline degradation product without chromatographic separation.

Despite the availability of several spectrophotometric methods for gliclazide determination, none has been specifically developed as a stability-indicating procedure capable of selectively resolving gliclazide from its official alkaline degradation product without chromatographic separation. The extensive spectral overlap between the parent drug and its degradation product represents a major analytical challenge that limits the applicability of conventional UV spectrophotometry. Therefore, there remains a need for a simple, selective, and environmentally sustainable analytical strategy capable of resolving this overlap while maintaining high analytical performance. The present work addresses this analytical gap through the complementary application of first-derivative spectrophotometry and Fourier Self-Deconvolution, providing a validated stability-indicating method suitable for routine pharmaceutical quality control.

## Experimental

### Instrument

A UV–Visible spectrophotometer (Jasco, Japan) equipped with quartz cells of 1.0 cm path length, the scanning speed was adjusted to 600 nm/min, with a bandwidth of 2 nm, slit width of 1 nm, and a data interval of 0.2 nm and interfaced with a computer operating the Jasco Spectrum Manager software for all spectrophotometric measurements. The absorption spectra of both the reference and sample solutions were recorded within the 200–400 nm wavelength range under optimized instrumental conditions.

Infrared (IR) spectra were recorded using a Fourier-transform infrared (FT-IR) spectrophotometer (Shimadzu FTIR-8400 S, Kyoto, Japan). Samples were prepared using the potassium bromide (KBr) disk technique and scanned over the range of 4000–400 cm⁻¹.

Mass spectrometric analysis was performed using an LC–MS system (Agilent 6420 Triple Quadrupole LC/MS, Agilent Technologies, Santa Clara, CA, USA) equipped with an electrospray ionization (ESI) source operating in positive ion mode. Samples were introduced after appropriate dilution with methanol. The mass spectra were recorded over a suitable m/z range to detect the molecular ion peaks of gliclazide (GLI) and its alkaline degradation product (IMP A).

### Materials, solvents and reagents

Gliclazide (GLI, purity 99.80%) was kindly supplied by Amoun Pharmaceutical Company (Cairo, Egypt). Diamicron tablets (labeled to contain 60 mg GLI; Batch No. 15GG407X) were obtained from Servier Pharmaceutical Company (Cairo, Egypt). Ethanol (HPLC grade), sodium hydroxide, and hydrochloric acid were purchased from Sigma-Aldrich (Germany). The alkaline degradation product of gliclazide (GLI impurity A) was prepared in-house according to the procedure described under the degradation study section.

### Preparation of Gliclazide impurity A (IMP A) (alkaline degradation product)

GLI (100 mg) was dissolved in 25 mL of 1 N ethanolic NaOH and refluxed at 100 °C for 1 h. Completion of degradation was confirmed by TLC (chloroform: methanol: ammonia, 2:8:0.5, v/v/v), showing GLI (Rf = 0.75) and IMP A (Rf = 0.50) with UV detection at 254 nm. The mixture was cooled, neutralized with 5 N HCl, evaporated to dryness, and purified by hot ethanol recrystallization. IMP A was identified by IR and MS spectroscopy. Preparative TLC was used for further purification, repeated as needed to obtain the desired amount.

### Stock solutions

#### Standard solutions

A standard stock solution of gliclazide (GLI) was prepared at a concentration of 1.0 mg/mL by dissolving an accurately weighed amount of the drug in ethanol. An appropriate aliquot of this stock solution was subsequently diluted with the same solvent to obtain a working standard solution of 100 µg/mL. All solutions were freshly prepared and demonstrated stability for at least four weeks when stored under refrigerated conditions.

#### Alkaline degradation product solutions

The standard stock solution of IMP A (1 mg/mL) was prepared by accurately weighing 100 mg of IMP A into a 100 mL volumetric flask and dissolving in ethanol. Standard working solutions of GLI and IMP A (100 µg/mL) were obtained by appropriate dilution of their respective stock solutions with ethanol.

### Preparation of laboratory-synthetic combinations

A series of calibration standards was prepared by transferring accurately measured aliquots equivalent to 2.5–20 µg of gliclazide (GLI) and 2.5–15 µg of its degradation product (IMP A) from their respective working standard solutions into separate 10 mL volumetric flasks. The volume in each flask was adjusted to the mark with ethanol, followed by thorough mixing to ensure homogeneity.

### Procedures

#### Linearity ranges and establishment of calibration graphs

To obtain final concentrations of 2.5–25 µg/mL for GLI and 2.5–15 µg/mL for IMP A by using six calibration points, appropriate aliquots from their respective standard working solutions (100 µg/mL) were transferred into two separate sets of 10 mL volumetric flasks and diluted to volume with ethanol. The absorption spectra of the prepared solutions were recorded over the wavelength range of 200–400 nm, using ethanol as a blank.

*First Derivative spectrophotometric method (*^*1*^*D)*.

The previously stored absorption spectra were processed to obtain the first derivative spectra, using ethanol as a blank. The derivative amplitudes were measured at 243 nm for GLI and 227 nm for IMP A. Calibration curves were constructed by plotting the measured signals of GLI (243 nm) and IMP A (227 nm) against their respective concentrations. The corresponding regression equations were then established.

*Fourier self-Deconvolution spectrophotometric method (FSD)*.

The stored spectra were processed using the Fourier Self-Deconvolution (FSD) technique integrated into the Jasco Spectrum Manager software. The deconvoluted spectral signals of GLI at 235 nm and IMP A at 224 nm were plotted against their respective concentrations. The resulting regression equations were subsequently applied to determine the amounts of GLI and IMP A in laboratory-prepared mixtures, as well as the GLI content in Diamicron tablets.

#### Quantification of Laboratory-Prepared Mixtures

The previously recorded spectra of the laboratory-prepared mixtures were processed as described in the “Linearity and Construction of Calibration Graphs” section for each analytical method. The concentrations of GLI and IMP A were then determined using the corresponding regression equations previously established.

#### Analysis of Diamicron tablets

Ten Diamicron tablets were accurately weighed and finely powdered. A portion of the powder equivalent to one tablet, containing 60 mg of gliclazide (GLI), was transferred into a 100 mL volumetric flask, followed by the addition of 30 mL of ethanol as a diluent. The mixture was sonicated for 30 min to ensure complete extraction of the drug, then diluted to volume with ethanol and mixed thoroughly. The obtained dispersion was filtered through a dry Whatman filter paper using a dry funnel, and the first few milliliters of filtrate were discarded. The resulting stock solution (0.6 mg/mL GLI) was further diluted with ethanol to obtain a working solution of 6 µg/mL. Additional dilutions were prepared in 100 mL volumetric flasks as required. The analytical procedures described under “Linearity and Construction of Calibration Graphs” were applied to determine the GLI concentration. Method accuracy and validity were verified by applying the standard addition technique, and the final GLI concentrations were calculated using the respective regression equations.

## Results and discussion

Compared with previously reported stability-indicating chromatographic methods, including UPLC^17^ and MEKC^20^, the proposed method offers a simpler analytical workflow, requires less expensive instrumentation, consumes less organic solvent, and is more suitable for routine quality-control laboratories where advanced chromatographic systems may not be readily available. Although chromatographic methods generally provide superior separation efficiency and broader impurity profiling, the present derivative spectrophotometric strategy successfully resolves gliclazide from its official alkaline degradation product without chromatographic separation through complementary mathematical signal processing. In comparison with previously reported stability-indicating spectrophotometric methods^23^, the proposed method demonstrates enhanced analytical performance by integrating Fourier Self-Deconvolution with first-derivative spectrophotometry, together with comprehensive validation and sustainability assessment, thereby providing a practical and reliable alternative for routine pharmaceutical analysis as illustrated in Table S1.

### Identification of the alkaline degradation product of GLI (IMP A)

Alkaline degradation was selected as the degradation pathway investigated in the present study because it reproducibly generated the official impurity of gliclazide and enabled the development of a stability-indicating analytical method. Alkaline hydrolysis resulted in significant and well-defined degradation, enabling the isolation and characterization of the major degradation product (IMP A).

**Fig. 2:**
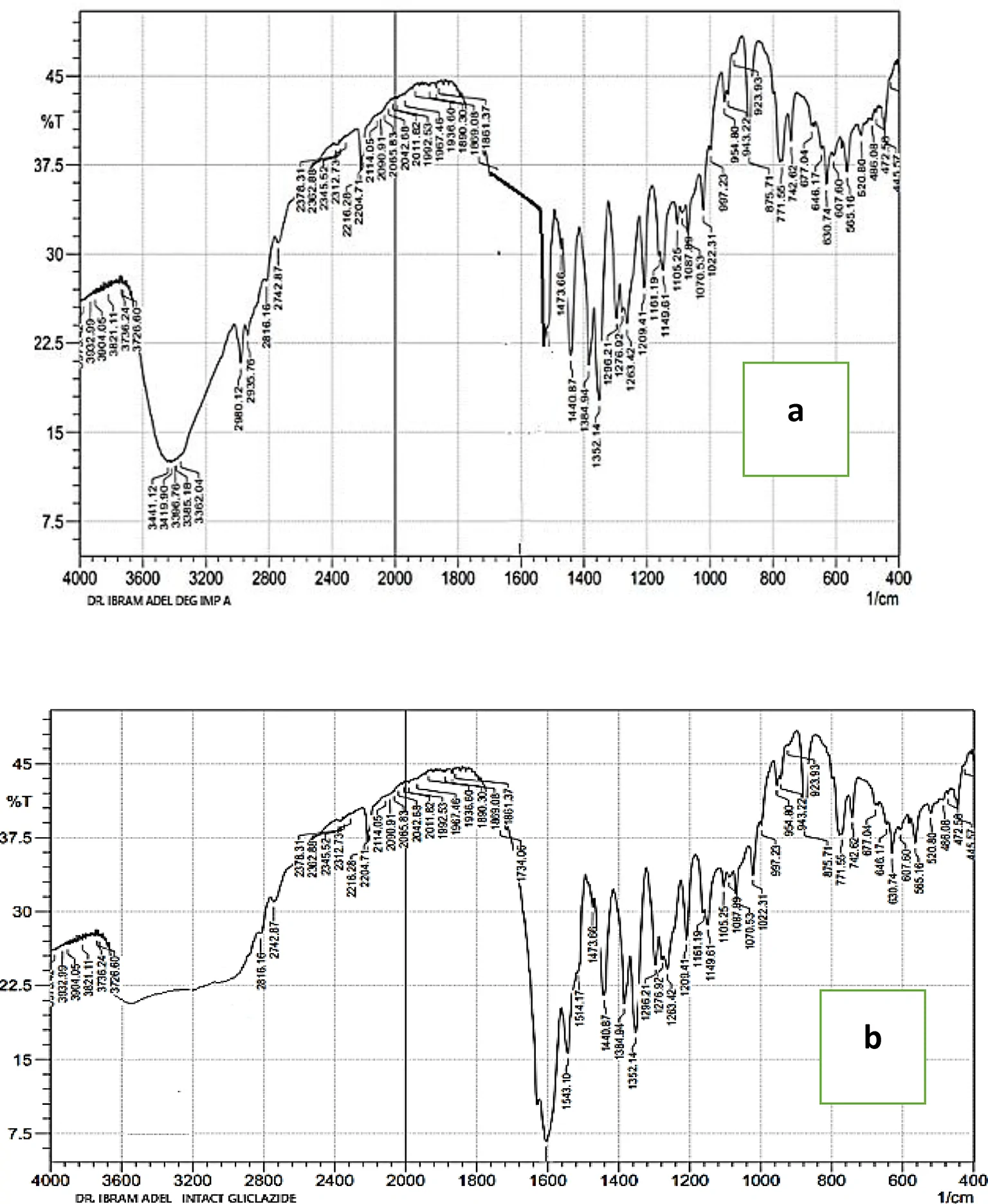

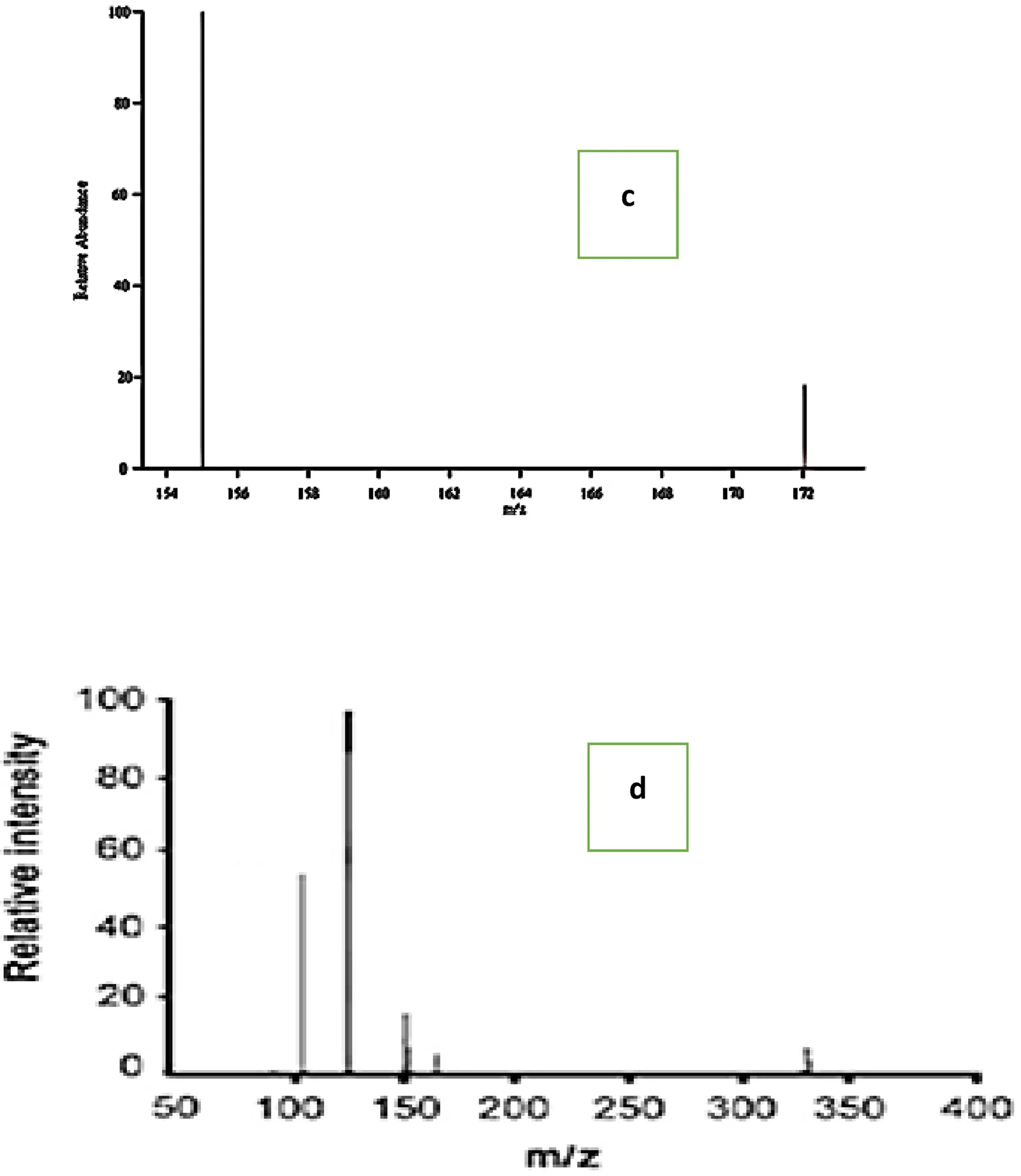
IR spectrum of IMP A (**a**), GLI (**b**), MS spectrum of IMP A (**c**), MS spectrum of GLI (**d**).

GLI was refluxed with 1 N ethanolic NaOH at 100 °C for 1 h to achieve complete degradation and obtain a high yield of the alkaline degradation product, IMP A. The refluxed solution was neutralized, evaporated to dryness, and the product was purified by extraction in hot ethanol followed by evaporation. TLC analysis revealed a distinct spot for IMP A (Rf = 0.50), different from that of intact GLI (Rf = 0.75). The IR spectrum of IMP A (Fig. 2(a)) showed the disappearance of the CO stretching band at 1650 cm⁻¹ ^24^, attributed to the carbonyl group present in the IR spectrum of intact GLI (Fig. 2(b)). Mass spectrometry further confirmed the identity of IMP A, showing an M⁺ peak at m/z 172 corresponding to its molecular weight (Fig. 2(c)), and an M⁺ peak at m/z 324 corresponding to GLI (Fig. 2(d)). These findings confirm that alkaline hydrolysis of the carbonyl group in the amide moiety of GLI resulted in the formation of IMP A. The combined IR and MS analyses verified the successful isolation of purified IMP A. This approach offers a cost-effective alternative to purchasing the compound, and the obtained IMP A, together with GLI and other antidiabetic agents, can be utilized in future research on type II diabetes mellitus management. The STABLE assessment^25^ yielded a score of 25 for gliclazide under alkaline conditions, indicating high degradation susceptibility and.

supporting the selection of alkaline stress as the most relevant degradation pathway in this study. The detailed evaluation is presented in Supplementary Fig. S1.

### Methods optimization and validation

**Fig. 3:**
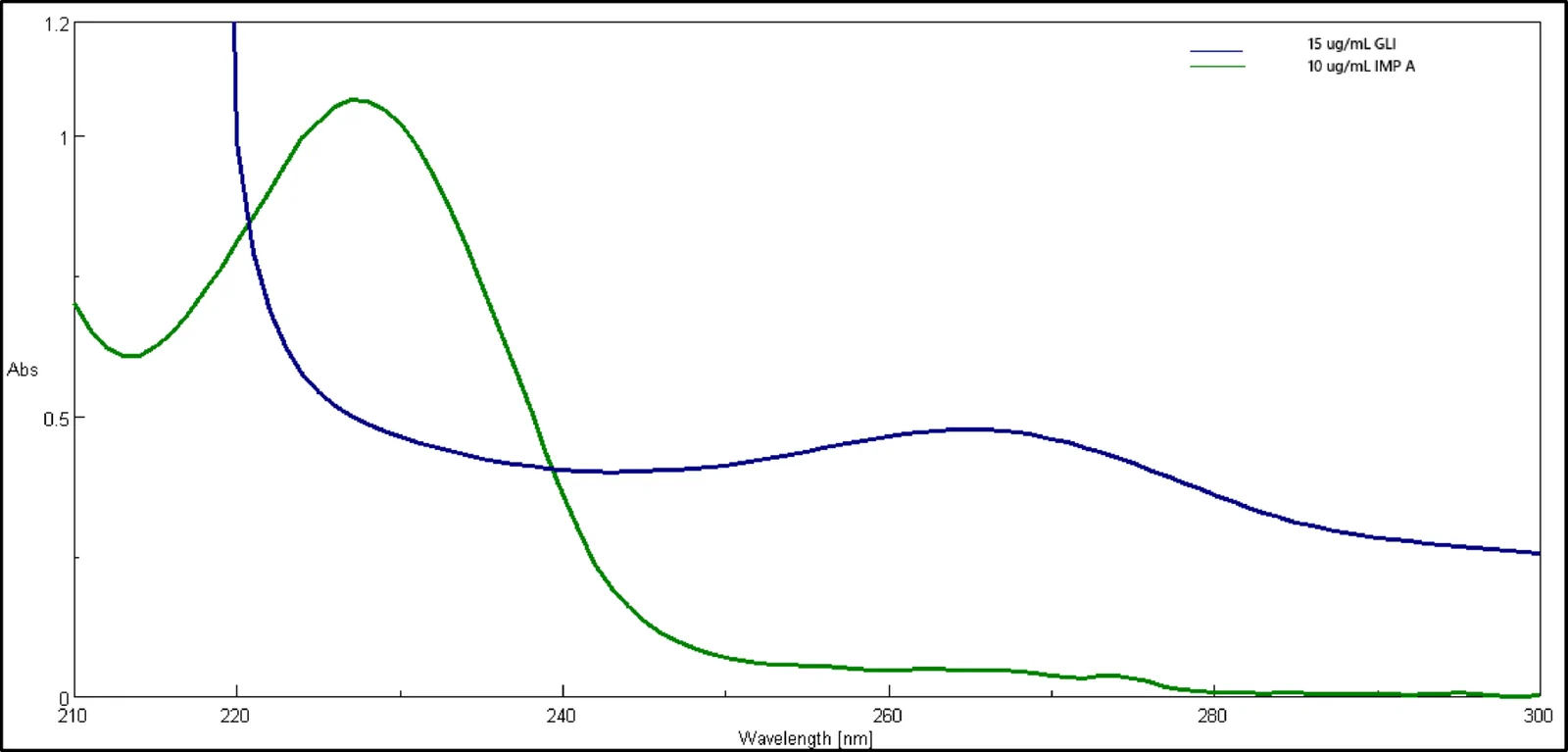
Absorption spectra of GLI (15 µg/ml) and IMPA (10 µg/ml) utilizing ethanol as a blank.

The absorption spectrum of GLI overlapped significantly with that of IMP A in the range of 210–280 nm, with no zero-crossing points observed for either compound (Fig. 3). The primary aim of the proposed methods is to resolve such severe spectral interference using simple and flexible spectrophotometric techniques, providing a cost- and time-efficient alternative to more laborious and expensive chromatographic methods. The developed techniques were validated and subsequently applied to the analysis of pharmaceutical dosage forms.

#### Methods **o**ptimization

*First derivative spectrophotometric method (*^1^*D)*.

**Fig. 4:**
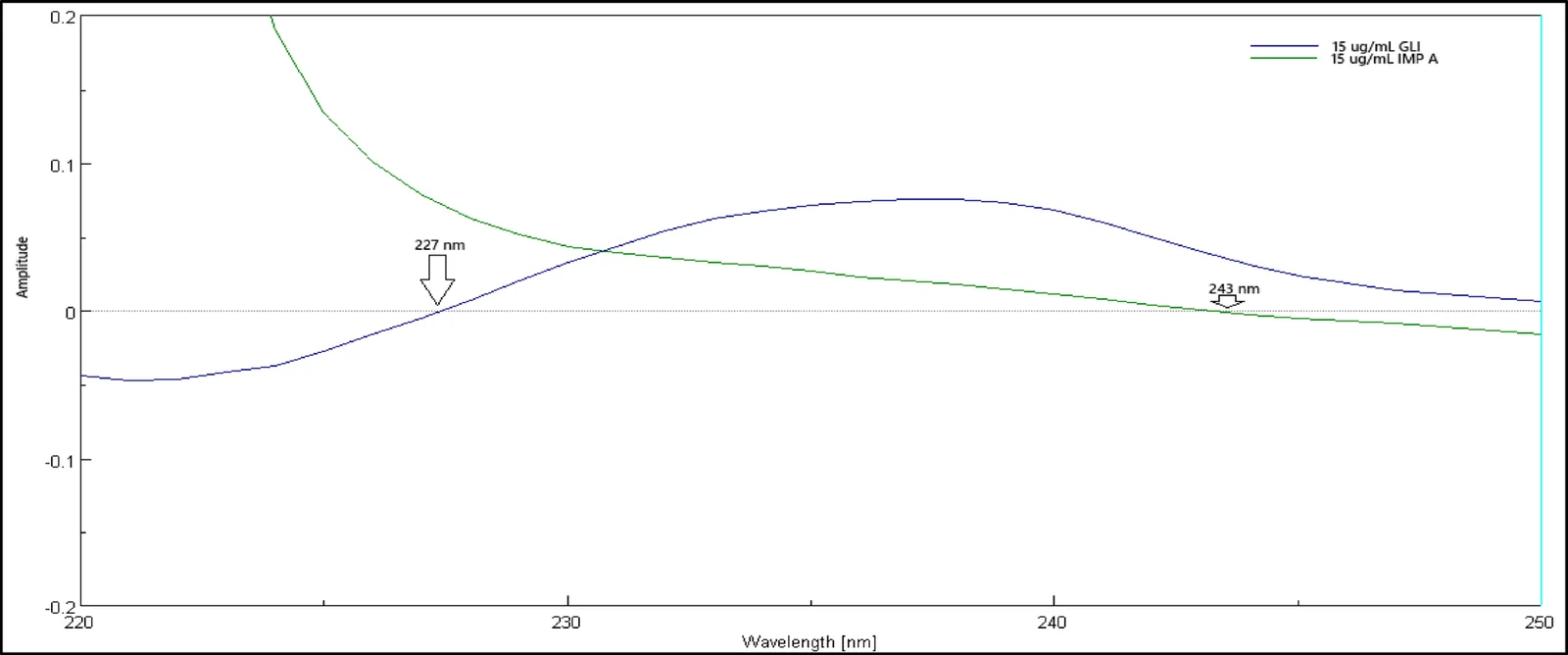
First derivative absorption spectra of GLI (15 µg/ml) showing no contribution of IMP A at 243 nm and IMP A (15 µg/ml) at 227 nm (zero-crossing point of IMP A).

In this method, the absorption spectra of GLI and IMP A were transformed into first derivative spectra using a wavelength interval (Δλ) of 5 nm and a scaling factor of 10. For GLI determination, 243 nm was selected as the measurement wavelength, corresponding to the zero-crossing point of IMP A (Fig. 4), and the calibration curve was established at 243 nm. For IMP A determination, amplitudes measured at 227 nm, the zero-crossing point of GLI, were plotted against the corresponding concentrations of IMP A. The resulting regression equations were then applied to quantify GLI and IMP A in laboratory-prepared mixtures (Fig. 4).

*Fourier self-Deconvolution spectrophotometric method (FSD)*.

Fourier self-deconvolution (FSD) is a widely used band-narrowing technique that facilitates resolving overlapping spectral bands, as in the case of GLI and IMP A. This method enables precise identification of the peak positions of each component in the spectra of GLI–IMP A mixtures, which consist of multiple overlapping bands of similar bandwidth.

**Fig. 5:**
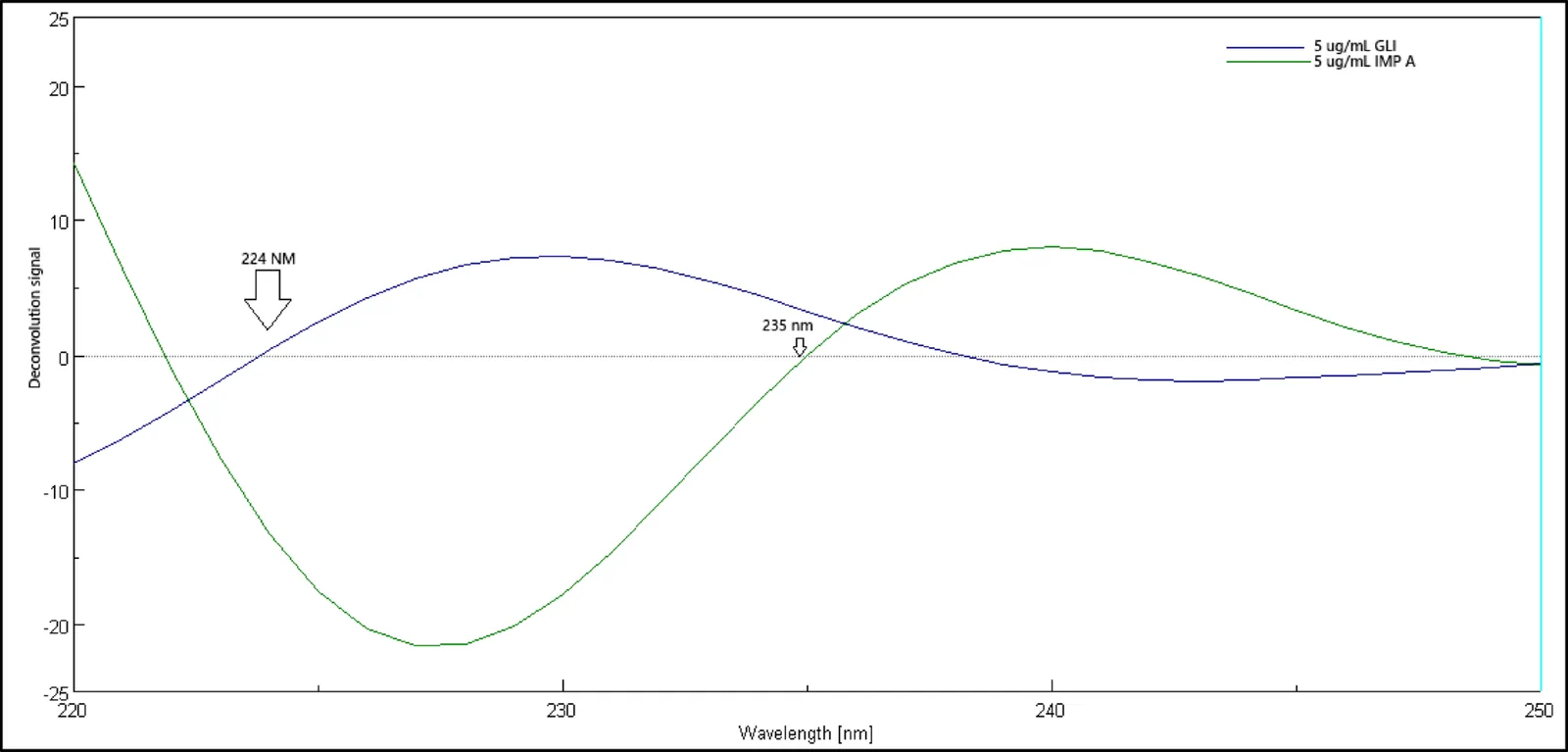
Deconvoluted spectra of GLI at 235 nm with a zero-crossing point for IMP A and IMP A at 224 nm with a zero-crossing point for GLI.

Both the extent of band narrowing achieved (effective narrowing) and the extent of signal-to-noise ratio deterioration resulting from FSD are strongly influenced by the choice of filter function^26^.. The shape of the deconvolved bands is also strongly influenced by the selected filter function. The stored absorption spectra of GLI and IMP A were resolved using the FSD function in the Jasco Spectrum Manager software. The resulting deconvoluted spectra were used to quantify GLI at 235 nm and IMP A at 224 nm (Fig. 5), each measured at the zero-crossing point of the other. A full width at half maximum (FWHM) value of 50, based on the expected Lorentzian waveform, was applied. Various FWHM values were tested to achieve well-resolved spectra with accurate and reproducible recoveries. The FWHM parameter is critical because the FSD program cannot resolve overlapping spectra when the peak bands have significantly different widths. Therefore, the half-widths of the spectral bands, both before and after deconvolution, were considered, as represented by their respective FWHM values^27,28^.

A direct comparison between the First Derivative (D¹) and Fourier Self-Deconvolution (FSD) approaches demonstrated the complementary analytical value of FSD as seen in in Table S2. While both methods achieved excellent linearity, accuracy, and sensitivity, FSD provided good spectral discrimination by enhancing the resolution of the highly overlapped absorption bands, allowing more selective measurements of both analytes. Moreover, FSD extended the linear range for gliclazide determination (2.5–25 µg mL⁻¹ versus 2.5–15 µg mL⁻¹) and yielded lower intra-day and inter-day RSD values for both gliclazide and IMP A, indicating improved method precision. These findings demonstrate that the contribution of FSD extends beyond mathematical signal processing by improving spectral resolution and strengthening the analytical performance of the proposed stability-indicating method.

#### Methods validation

ICH guidelines and criteria were followed upon developing the current approaches^29^. Table 1 summarizes all regression results. According to ICH Q2(R1) guidelines, acceptable precision for assay methods should demonstrate a %RSD not exceeding 2%. The obtained %RSD values for repeatability and intermediate precision were all below 2%, confirming the adequate precision of the proposed method. The selectivity of the proposed methods was demonstrated by the quantitative determination of GLI and IMP A in laboratory-prepared mixtures (Table 2). Furthermore, the methods were successfully applied to the assay of GLI in Diamicron tablets without interference from excipients, as confirmed by the results of the standard addition technique (Table 3).

**Table 1:** Regression and validation parameters of the current spectrophotometric approaches for the concurrent estimation of GLI and IMP A.

Drug	GLI		IMP A	
Validation parameters	FSD	^1^D	FSD	^1^D
Linearity Range (µg/mL)	2.5–25		2.5–15	
Regression coefficient (r)	0.9998	0.9999	0.9997	0.9997
Slope	0.1575	0.0026	0.8583	0.0029
Intercept	0.0196	0.0002	0.1729	0.0047
g/mL)µ) LOD	0.43	0.31	0.40	0.39
g/mL)µ) LOQ	1.31	0.94	1.21	1.20
Accuracy (Mean^a^ ± SD)	99.48 ± 0.83	100.87 ± 1.52	99.06 ± 1.23	101.28 ± 1.81
Intra-day precision (RSD %)^b^	0.59	0.63	0.52	0.87
Inter-day precision (RSD %) ^b^	1.42	1.68	0.98	1.65

^a^ Average of nine estimations.

^b^ RSD% of nine estimations.

**Table 2:** Concurrent estimation of GLI and IMP A in their laboratory-synthetic combinations applying the current spectrophotometric approaches.

Conc. Taken (µg/mL)		% Recovery ^a^			
GLI	IMP A	GLI		IMP A	
		FSD	^1^D	FSD	^1^D
2.5	2.5	101.26	99.25	101.65	100.29
6	6	100.22	100.73	100.81	101.29
10	10	98.28	99.82	100.28	98.76
15	10	100.39	98.29	100.92	98.82
25	15	101.29	99.92	99.63	99.92
Mean ± SD		100.28 ± 1.22	99.60 ± 0.90	100.65 ± 0.75	99.81 ± 1.06

^a^ Average of three determinations.

**Table 3:** Applying the current spectrophotometric approaches for analyzing GLI in Diamicron tablets and application of the standard addition technique.

Conc. Taken (µg/ml)		% Recovery ^a^			
		GLI			
		FSD		^1^D	
Tablet	Added	Tablet	Added	Tablet	Added
	3		100.46		99.77
6	6	100.44	98.95	100.34	100.82
	9		100.28		101.11
Mean		100.44	99.89	100.34	100.56
SD		1.53	0.82	0.89	0.70

^a^Average of three determinations.

### Statistical analysis

A statistical comparison between the results of the proposed methods and those of the reference method is presented in Table 4. The calculated t and F values were lower than the corresponding theoretical values, indicating no significant difference in accuracy or precision between the developed and reported procedures. These findings confirm that the proposed methods can quantitatively determine GLI with high precision and acceptable accuracy.

**Table 4:** Statistical data interpretation and comparison of the results produced by establishing the current spectrophotometric approaches and the reference method for the estimation of GLI in the pharmaceutical dosage form.

Form	Parameters	Reference method **	GLI	
			FSD	^1^D
Pharmaceutical dosage form	Mean	101.75	100.44	100.34
	SD	1.68	1.53	0.89
	Variance	2.832	2.341	0.792
	t-test*	---	1.14	1.81
	F-ratio*	---	1.21	3.58

* The theoretical values of t and F at *P* = 0.05 are (2.23) and (5.05), respectively where *n* = 6.

**Reference HPLC method^15^: For the determination of GLI consisting of Isocratic elution at a flow rate of 1.2 ml min − 1 was employed on a symmetry C18 column at ambient temperature. The mobile phase consisted of methanol: phosphate buffer 50:50 (V/V). The UV detection wavelength was at 210 nm.

Student’s t-test was applied at a 95% confidence level with the corresponding degrees of freedom (df = n₁ + n₂ − 2).

### One-way ANOVA

The one-way ANOVA procedure was conducted to make a comparison between the mean recoveries of the suggested spectrophotometric methods. The results of the data are displayed in Table 5. Statistical comparison between the proposed methods was performed using one-way ANOVA at a 95% confidence level (α = 0.05).

**Table 5:** One-way ANOVA test for the determination of GLI by the current spectrophotometric methods.

GLI					
Source of Variation	Sum of squares	Degrees of freedom	Mean square	F-value (3.88)*	P-value
Between groups	0.16	2	0.08	0.08	0.920
Within groups	11.82	12	0.98		
total	11.98	14			

* Theoretical F-value.

### Assessment of greenness of the proposed methods

Green chemistry has become an essential paradigm in modern analytical laboratories, emphasizing the need to minimize the environmental footprint of analytical procedures. In the present study, the environmental sustainability of the proposed spectrophotometric methods was comprehensively assessed considering four key parameters: the quantity and inherent hazards of the chemicals used, energy consumption, occupational and safety risks, and waste generation. The greenness evaluation was performed employing four recognized metrics — the National Environmental Methods Index (NEMI), the Analytical GREEnness metric (AGREE), the Green Analytical Procedure Index (GAPI), and the Analytical Eco-Scale — to provide a multidimensional and holistic insight into the environmental performance of the developed method. The first system is principally based on the National Environmental Methods Index (NEMI)^30^, where the greenness is designated by a pictogram divided into four quadrants. The NEMI is a qualitative technique in which pictograms indicate whether hazardous or corrosive reagents are applied or whether the process generates considerable volumes of waste.

**Fig. 6:**
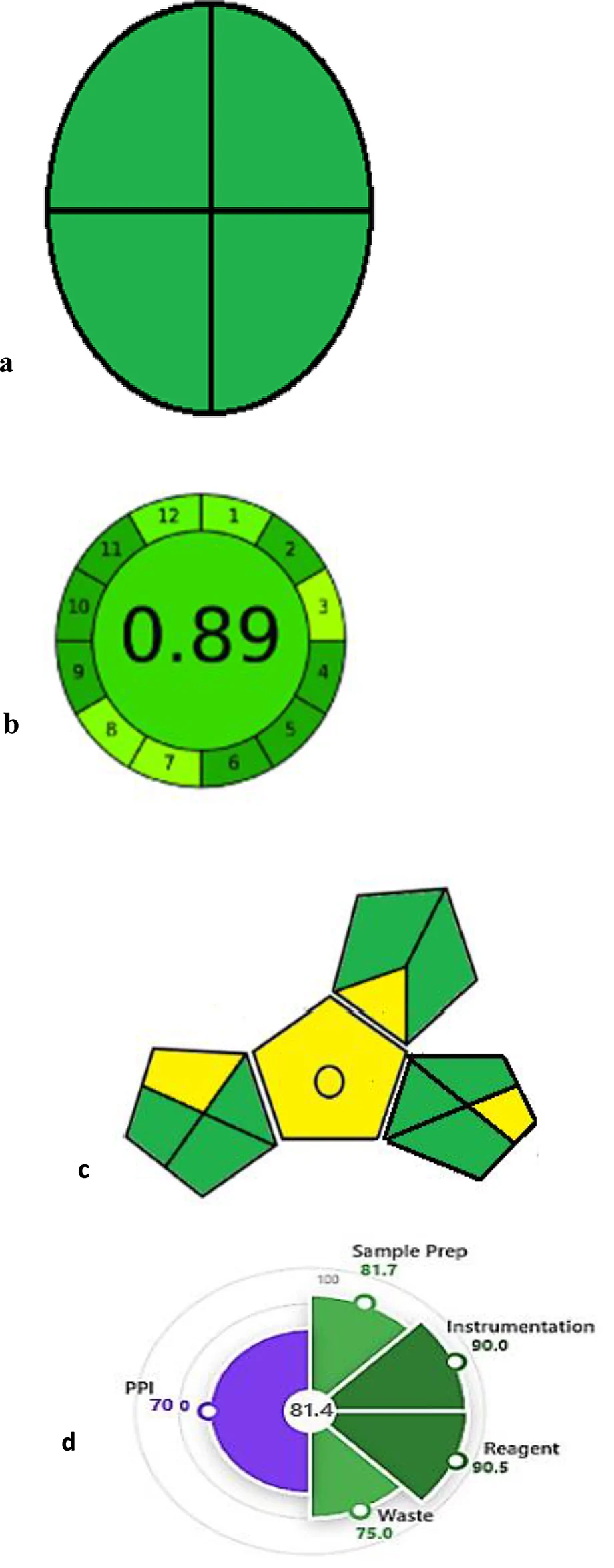

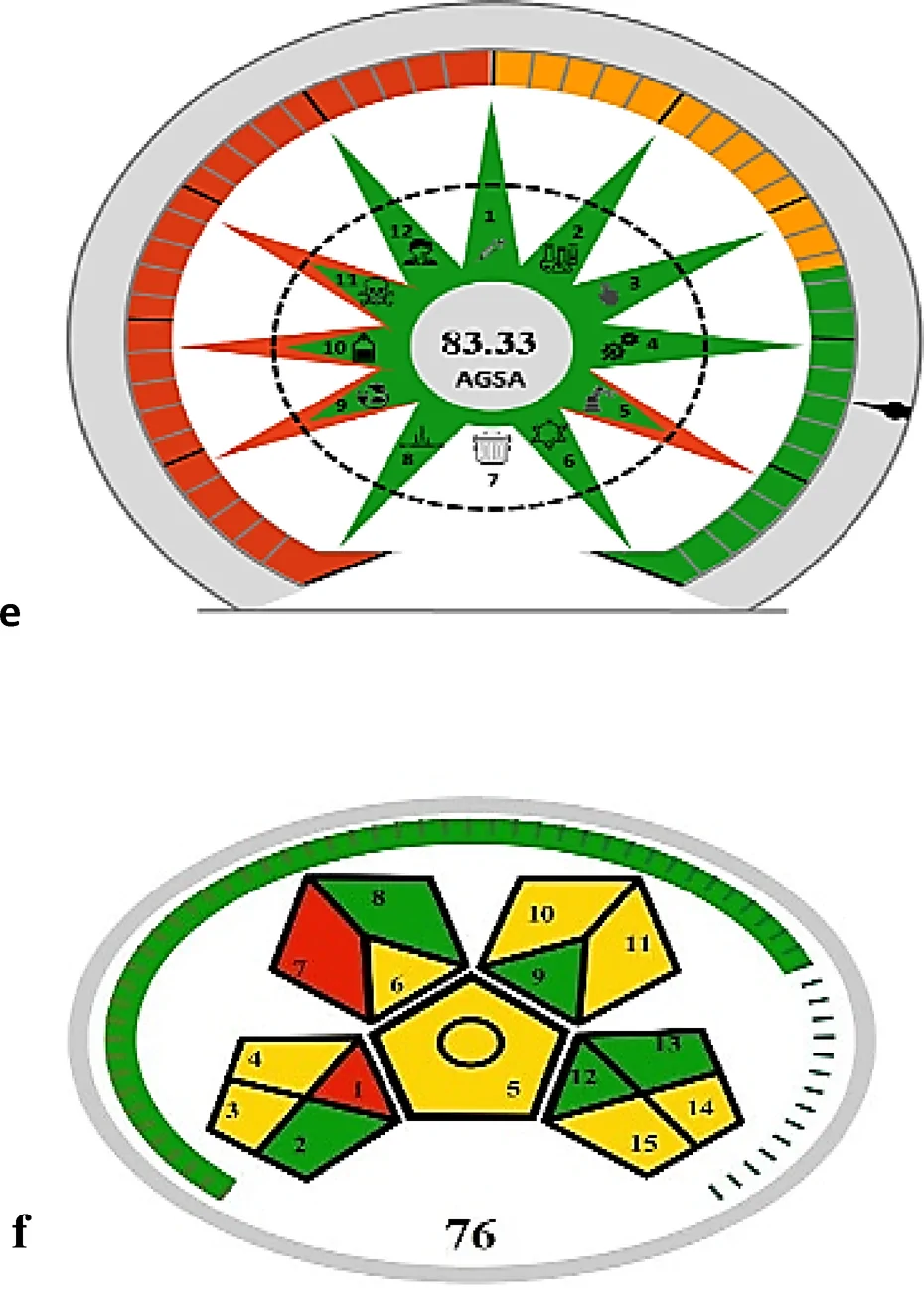
Assessment of greenness of the current spectrophotometric approaches utilizing NEMI method (a), AGREE calculator (b), GAPI index (c), EPPI evaluation (d), AGSA tool, and MOGAPI method.

According to the National Environmental Methods Index (NEMI), the four quadrants representing the proposed spectrophotometric methods (Fig. 6a) were fully shaded in green, indicating the eco-friendly nature of the developed procedure. This classification is attributed to the exclusive use of ethanol as a solvent, which is neither persistent, bioaccumulative, nor toxic (PBT), and is considered a low-hazard, renewable solvent. Moreover, the analytical pH range of 2–12 falls within the environmentally acceptable limits, and the total waste generated per analysis was estimated to be less than 50 g, further confirming the minimal environmental burden of the method. The AGREE program forms the backbone of the second system^31–33^,^34^. The 12 principles of Green Analytical Chemistry (GAC) form the basis of the clock-shaped AGREE graph. The method’s compliance with these principles is visually represented on a color scale (red–yellow–green), with each sector corresponding to one principle. At the center of the graph, a combined color and a numerical score (ranging from 0 to 1) indicate the overall greenness rating. The AGREE tool evaluates the environmental impact of several factors, including reagent toxicity, waste generation, energy consumption, labor intensity, and degree of automation, with the aim of minimizing environmental harm and enhancing sustainability. In this study, the calculated AGREE score was 0.89, indicating a high level of eco-friendliness for the proposed method (Fig. 6b).

The green analytical procedure index, GAPI^35–37^ was also utilized to evaluate green assessment. GAPI is composed of fifteen separate areas (five pentacle shapes) for estimating and evaluating the environmental impact of each step in any analytical process, from sample preparation to instrumentation and waste quantity and treatment. All of these parameters are displayed on the pentagram and are colored green, yellow, or red to indicate a minimum, medium or high impact on the environment, respectively.

The developed spectroscopic procedures incorporated in-line sample preparation, eliminating the need for intermediate storage or transfer steps. Samples were prepared directly in the analytical medium, without extraction, derivatization, or the use of hazardous reagents. Ethanol, a green solvent, was employed throughout the process, minimizing environmental and occupational risks.

In the AGREE evaluation, Sects. 4 and 9 were highlighted in yellow, indicating the use of conventional storage conditions and a moderate total solvent consumption between 10 and 100 mL, respectively (Fig. 6c). Similarly, Sect. 5 was colored yellow due to the quantification and filtration steps required during sample preparation for the pharmaceutical dosage form. In addition, Sect. 14 was assigned a yellow color, reflecting a waste volume slightly exceeding 10 mL (Fig. 6c).

Despite these minor yellow zones, the overall GAPI pictogram demonstrated a predominantly green profile, confirming that the proposed spectroscopic procedures are environmentally benign and align well with the principles of Green Analytical Chemistry (GAC). The analytical Eco-Scale^36,38^ was also utilized to evaluate the greenness of the current spectrophotometric approaches for the concurrent analysis of GLI and IMP A. This tool is a comprehensive current scale that calculates the penalty points (PPs) for any process’s analytical parameters, such as the nature and quantity of reagents, occupational hazards, the energy required, and the waste generated. The calculated penalty points will be subtracted from 100 (the ideal green score). At higher scores, the analytical process is more cost-effective and environmentally friendly (near 100), (Table 6). Due to the minimal amount of waste and the use of less hazardous chemicals, the PPs score in this study was assessed to be eight, and the analytical eco-scale score is 92, demonstrating outstanding green behavior, as shown in Table 6.

**Table 6:** Greenness assessment of the proposed spectrophotometric methods according to the analytical Eco-scale method.

Parameters	Penalty points
Ethanol	2
Instrument	
Energy consumption^a^	0
Occupational hazard	0
Waste (1–10 ml, no treatment)^b^	6
Total penalty points	8
Analytical EcoScale total score^c, d^	92
Comment	Excellent green analysis

^a^ A score of 0 is given for UV/is spectrophotometer; the energy utilized is ≤ 0.1 kWh per sample.

^b^ All reported waste volumes refer to per-sample analysis, not to the total experimental waste generated during method development.

^c^ Analytical Eco-Scale total score = 100- total penalty point.

^d^ If the score is > 75, it shows excellent green analysis.

If the score is > 50, it shows an acceptable green analysis.

If the score is < 50, it shows inadequate green analysis.

In the AGREE evaluation, most parameters exhibited a strong green profile. Sections 4 and 9 were colored yellow, reflecting the use of conventional storage conditions and a total solvent consumption ranging from 10 to 100 mL, respectively (Fig. 6c). Similarly, Sect. 5 was marked yellow due to the quantifi6cation stage involving simple filtration during the analysis of the pharmaceutical dosage form. Section 14 also appeared yellow, corresponding to the generation of waste volumes slightly above 10 mL (Fig.6c).

Despite these minor exceptions, the overall GAPI assessment confirmed that the proposed spectrophotometric approaches demonstrate a high degree of environmental sustainability, balancing analytical efficiency with minimal ecological footprint^37^.

In light of recent sustainable analytical frameworks, the proposed method was qualitatively evaluated using the Environmental, Performance, and Practicality Index (EPPI)^39^. From an environmental perspective, the method employs aqueous media with minimal solvent consumption and generates low per-sample waste without the use of toxic reagents or energy-intensive steps. In terms of analytical performance, the method demonstrated excellent linearity, accuracy, precision (%RSD < 2%), and enhanced selectivity through the integration of first derivative and FSD techniques. Regarding practicality, the proposed approach is simple, cost-effective, rapid, and relies on widely available UV spectrophotometric instrumentation, making it suitable for routine quality control laboratories. Collectively, these attributes confirm that the proposed method aligns well with the core principles of EPPI and represents a sustainable and practical analytical alternative (Fig. 6d).

Established criteria and assigned a numerical score reflecting its adherence or impact. The cumulative scores generate the Analytical Green Sustainability Assessment (AGSA), which provides a quantitative measure of an analytical method’s overall environmental sustainability and compliance with green chemistry principles. The evaluation considers factors such as sample preparation, reagent toxicity, waste generation, energy consumption, automation, and operator safety.

Applying this approach to the present method, minimal sample treatment was required no extensive solid-phase extraction, liquid–liquid extraction, or protein precipitation using a small sample volume. Measurements were conducted in methanol. The method employed low-energy instruments and low-toxicity reagents, with safe procedures supported by standard personal protective equipment (Fig. 6e).

The AGSA score of 83.33 highlights the excellent greenness and sustainability of the proposed analytical approach. This tool can efficiently assess the ecological footprint of analytical methods, and future work will validate AGSA across published methods to confirm its consistency and correlation with other green metrics. Community input is also planned to refine and standardize the metric, promoting broader acceptance and cross-disciplinary comparison^40^.

Every stage of the analytical procedure, including sample preparation, sample volume, solvent and reagent safety, instrumentation, and waste management, was evaluated using the Green Analytical Procedure Index (GAPI), which encompasses fifteen assessment criteria. In this system, green, yellow, and red colors indicate low, moderate, and high environmental impacts, respectively. However, GAPI alone lacks a unified quantitative scale for comparing different analytical methods. To overcome this limitation, the Modernized Green Analytical Procedure Index (MoGAPI) integrates the numerical scoring approach of the Analytical Eco-Scale with the visual interpretability of GAPI, thus providing a more comprehensive assessment of a method’s environmental sustainability. As illustrated in Fig. 6f, the proposed analytical method exhibited excellent environmental performance, achieving a MoGAPI score of 76, indicative of strong adherence to green chemistry principles^41^.

A comparative evaluation of the proposed method with previously reported analytical techniques for the determination of gliclazide was also conducted in accordance with the principles of Green Analytical Chemistry (GAC). Conventional chromatographic methods, such as HPLC and LC–MS, although highly sensitive and selective, typically involve the use of large volumes of hazardous organic solvents (methanol and acetonitrile), require high energy consumption, and generate considerable chemical waste. In contrast, the proposed spectrophotometric methods utilize ethanol as a green solvent, consume minimal energy, and produce significantly lower waste, thus reducing their environmental footprint.

Moreover, the simplicity of the instrumentation and the absence of complex sample preparation steps further enhance the practicality and sustainability of the proposed approach. Despite its simplicity, the method maintains excellent analytical performance in terms of accuracy, precision, and selectivity. Therefore, the proposed method represents a greener and more sustainable alternative to conventional analytical techniques, aligning well with the core principles of GAC.

### Multi-Color Analytical Profile Assessment of the proposed techniques

The aspects of the analytical approach, such as precision, accuracy, applicability, sensitivity, the quantity of waste, reagent toxicity, other dangers, sample throughput, cost analysis and sample energy consumption, are evaluated using cutting-edge technologies. We have been trying to implement these tactics for some time now, and as part of that process, we have been evaluating several analytical tools, including the hexagonal tool and the RGB12 models^42^. The RGB 12 model, which adheres strictly to WAC guidelines, is also provided in a spreadsheet format^43^. Yet, the hexagonal instrument relies on the concept of penalization. Both the comprehensive evaluation tables and the final ranking table of the criteria in terms of the ranges of penalty points are included in the original publication^44^. First, the adopted method was tested with a hexagonal tool. In Fig. S2, the results for the hexagon tool are shown as scores of 0, 1, 2, 3, and 4. These scores correlate to the analytical approach evaluations of excellent, satisfactory, average, inadequate, and unsuccessful. Four zeros and three ones were the outcomes of the present spectrophotometric method. This proved that the existing spectrophotometric methods were both sustainable and satisfied all analytical requirements, demonstrating great environmental friendliness.

The Click Analytical Chemistry Index (CACI)^45^ was applied to evaluate the practicality and applicability of the proposed method. The obtained CACI score was 80%, indicating that the method is highly practical, simple, and suitable for routine analytical applications Fig. S3.

The Carbon Footprint Reduction Index (CaFRI) was applied to quantitatively assess the environmental impact of the proposed spectrophotometric method. The evaluation considers key factors such as energy consumption, CO₂ emissions, reagent usage, waste generation, transportation, and personnel. The obtained CaFRI score of 86 indicates a low carbon footprint, reflecting the method’s high environmental sustainability. This favorable score can be attributed to the use of low-energy instrumentation, minimal solvent consumption, simplified sample preparation, and reduced waste generation^46^. Overall, the results demonstrate that the proposed method not only maintains analytical performance but also contributes significantly to sustainable and green analytical practices Fig. S4.

The Red Analytical Performance Index (RAPI)^47^ was applied to evaluate the analytical performance of the proposed method through a comprehensive assessment of key validation parameters, including accuracy, precision, linearity, sensitivity, selectivity, robustness, and applicability. The obtained RAPI score was 32.5, reflecting the overall analytical performance profile of the developed spectrophotometric method. Despite the inherent limitations associated with spectrophotometric techniques compared with more sophisticated chromatographic approaches, the proposed method demonstrated satisfactory validation characteristics and fulfilled the requirements for its intended application. The method exhibited acceptable accuracy, precision, and selectivity for the simultaneous determination of gliclazide and its alkaline degradation product, confirming its suitability for routine quality control analysis. The detailed RAPI assessment is presented in Supplementary Fig. S5.

The Analytical Method Reliability Index (AMRI)^48^ was employed to assess the overall reliability and fitness-for-purpose of the proposed analytical method. This index integrates critical validation characteristics, including accuracy, precision, selectivity, linearity, robustness, sensitivity, and successful application to real samples, into a single quantitative measure of method reliability. The obtained AMRI score of 83% demonstrates a high level of reliability and confirms the suitability of the proposed spectrophotometric method for routine quality control applications. The favorable score reflects the method’s consistent analytical performance, reproducibility, and ability to generate reliable results in the presence of the alkaline degradation product. The detailed AMRI evaluation is presented in Supplementary Fig. S6.

As shown in Table S3, the proposed method offers several advantages over previously reported spectrophotometric methods for gliclazide determination. Unlike the reported methods of Leleka and Vasyuk^12^and Aantakli^13^et al., which were developed for the determination of intact gliclazide only, the present method enables the simultaneous determination of gliclazide and its alkaline degradation product, demonstrating its stability-indicating capability. Furthermore, the proposed method combines satisfactory analytical performance with enhanced sustainability and reliability, as evidenced by the obtained AGSA, CaFRI, RAPI, and AMRI scores. These features highlight its suitability for routine quality-control laboratories while supporting the principles of modern sustainable analytical chemistry.

## Conclusion

The present study introduces simple, sensitive, and highly reproducible spectrophotometric methods for the simultaneous determination of gliclazide (GLI) and its alkaline degradation product (IMP A) using low-cost, eco-friendly solvents. Gliclazide continues to serve as a cornerstone in the pharmacotherapy of type II diabetes mellitus, and maintaining its stability and purity is essential to ensuring therapeutic efficacy and patient safety.

The proposed approaches effectively resolve the spectral overlap between GLI and its degradation product without the need for prior separation, offering a rapid and reliable analytical alternative to chromatographic techniques. Moreover, the methods demonstrate strong potential for extension to other multicomponent pharmaceutical systems where spectral interferences are encountered.

From an environmental standpoint, the greenness profile of the developed procedures was comprehensively assessed using six complementary evaluation tools, all of which confirmed the sustainability and low ecological footprint of the proposed methods.

Overall, these findings establish the developed UV-spectrophotometric techniques as robust, sustainable, and analytically sound options for routine quality control of gliclazide and related compounds in pharmaceutical analysis.

## Supplementary Information

Below is the link to the electronic supplementary material.

Supplementary Material 1

## Data Availability

All data obtained during this study are included in this published article.
