# Supplementary material for: Sustainable stability-indicating spectra manipulations for the concurrent quantification of gliclazide and its impurity A as the possible alkaline degradation product: Multi-Color Analytical Profile Assessment
Source: Sci Rep. 2026 Aug 22;16:26367. doi: 10.1038/s41598-026-67045-4 (PMC13499797; doi:10.1038/s41598-026-67045-4)
Supplement: Supplementary file 1 [file 41598_2026_67045_MOESM1_ESM.docx]

**Supplementary material**

**For**

**Sustainable Stability-Indicating Spectra Manipulations for the Concurrent Quantification of Gliclazide and its Impurity A as the Possible Alkaline Degradation Product: Green Profile Assessment**

**Ebraam B. Kamel^*1^, Ahmed Elsayed Abdelmegeed^2^, Hanaa Saleh^2^, Magda Elhenawee^2^,**

**Eman A. Bahgat^2^**

Fig. S1: STABLE-based assessment of gliclazide degradation behavior highlighting the susceptibility under alkaline conditions.

Fig. S2. Hexagon profile of the developed spectrophotometric methods.

Fig. S3. Click Analytical Chemistry Index (CACI) pictogram illustrating the practicality and applicability of the proposed spectrophotometric method

Fig. S4. Carbon Footprint Reduction Index (CaFRI) assessment of the proposed analytical method. The assessment was performed using the CaFRI web-based tool, a sustainability assessment platform designed to evaluate the carbon-footprint reduction potential of analytical methods. The figure was generated/exported from the CaFRI web tool. Available at: https://bit.ly/CaFRI.

Fig. S5. Red Analytical Performance Index (RAPI) pictogram illustrating the analytical performance assessment of the proposed method based on key validation parameters.

Fig. S6. Figure S6. Analytical Method Reliability Index (AMRI) assessment of the proposed analytical method. The assessment was performed using the AMRI web-based scoring tool/software, which provides a quantitative evaluation of analytical method reliability. The figure was generated/exported from the AMRI tool. Available at: http://bit.ly/AMRI2026.

Table S1. Critical comparison between the proposed method and previously reported stability-indicating methods for gliclazide determination.

| Method | UPLC^17^ | MEKC^20^ | Previous Spectrophotometric^23^ | Proposed |
| --- | --- | --- | --- | --- |
| Stability-indicating | ✓ | ✓ | ✓ | ✓ |
| Instrumentation | Advanced | Advanced | Simple | Simple |
| Sample preparation | Moderate | Moderate | Simple | Simple |
| Organic solvent consumption | High | Moderate | Low | Low |
| Cost | High | High | Low | Low |
| Routine QC suitability | Moderate | Moderate | Good | Excellent |
| Mathematical resolution | No | No | Limited | FSD + D¹ |
| Sustainability | Moderate | Moderate | Good | Excellent |

Table S2. **Comparative analytical performance of the First Derivative (D¹) and Fourier Self-Deconvolution (FSD) methods for the determination of gliclazide and its official impurity.**

| Parameter | D¹ | FSD | Advantage of FSD |
| --- | --- | --- | --- |
| Spectral resolution | Good | Improved | Better peak discrimination |
| GLI linear range | 2.5–15 | 2.5–25 | Wider working range |
| GLI Intra-day RSD | 0.63 | 0.59 | Better precision |
| GLI Inter-day RSD | 1.68 | 1.42 | Better precision |
| IMP A Intra-day RSD | 0.87 | 0.52 | Better precision |
| IMP A Inter-day RSD | 1.65 | 0.98 | Better precision |

Table S3. Comparison of the proposed method with reported spectrophotometric methods for gliclazide determination

| Parameter | Proposed Method | Leleka & Vasyuk (2022) | Aantakli et al. (2023) |
| --- | --- | --- | --- |
| Analytical target | GLI + alkaline degradation product | GLI only | GLI only |
| Stability-indicating capability | Yes | No | No |
| Simultaneous determination | Yes | No | No |
| Sample preparation | Simple, direct | Derivatization with BCG | Ion-pair complex formation |
| Solvent consumption | Low | Acetone | Toluene |
| Selectivity toward degradation product | Excellent | Not evaluated | Not evaluated |
| Linearity range (µg mL⁻¹) | 2.5–25 µg/mL | 62–94 µg/mL | 4.85–113.19 µg/mL |
| LOD | 0.43 µg/mL | ~4.02 µg/mL | 0.47 µg/mL |
| Application to degradation studies | Yes | No | No |
| RAPI score | 32.5 | 30 | 22 |
| AMRI score (%) | 83 | 60 | 75 |
| AGSA score | 69.44 | 62.76 | 60.43 |
| CaFRI score | 86 | 72 | 77 |
| CACI score | 80 | 70 | 65 |
